# Life Expectancy with and without Cognitive Impairment in Seven Latin American and Caribbean Countries

**DOI:** 10.1371/journal.pone.0121867

**Published:** 2015-03-23

**Authors:** Kimberly Ashby-Mitchell, Carol Jagger, Tony Fouweather, Kaarin J. Anstey

**Affiliations:** 1 Centre for Research on Ageing, Health and Wellbeing, The Australian National University, Australia; 2 Institute for Ageing and Health, Newcastle University, United Kingdom; Fundacion Huesped, ARGENTINA

## Abstract

**Background:**

The rising prevalence of cognitive impairment is an increasing challenge with the ageing of our populations but little is known about the burden in low- and middle- income Latin American and Caribbean countries (LAC) that are aging more rapidly than their developed counterparts. We examined life expectancies with cognitive impairment (CILE) and free of cognitive impairment (CIFLE) in seven developing LAC countries.

**Methods:**

Data from The Survey on Health, Well-being and Ageing in LAC (N = 10,597) was utilised and cognitive status was assessed by the Mini-Mental State Examination (MMSE). The Sullivan Method was applied to estimate CILE and CIFLE. Logistic regression was used to determine the effect of age, gender and education on cognitive outcome. Meta-regression models were fitted for all 7 countries together to investigate the relationship between CIFLE and education in men and women at age 60.

**Results:**

The prevalence of CI increased with age in all countries except Uruguay and with a significant gender effect observed only in Mexico where men had lower odds of CI compared to women [OR = 0.464 95% CInt (0.268 – 0.806)]. Low education was associated with increased prevalence of CI in Brazil [OR = 4.848 (1.173–20.044)], Chile [OR = 3.107 (1.098–8.793), Cuba [OR = 2.295 (1.247–4.225)] and Mexico [OR = 3.838 (1.368–10.765). For males, total life expectancy (TLE) at age 60 was highest in Cuba (19.7 years) and lowest in Brazil and Uruguay (17.6 years). TLE for females at age 60 was highest for Chileans (22.8 years) and lowest for Brazilians (20.2 years). CIFLE for men was greatest in Cuba (19.0 years) and least in Brazil (16.7 years). These differences did not appear to be explained by educational level (Men: p = 0.408, women: p = 0.695).

**Conclusion:**

Increasing age, female sex and low education were associated with higher CI in LAC reflecting patterns found in other countries.

## Introduction

Cognitive impairment (CI) is a condition where a person has trouble remembering, learning new things, concentrating or making decisions that affect their everyday life [1]. CI can be mild or can be severe enough to affect an individual’s ability to live independently [1]. The rising prevalence of CI is an increasing challenge with the ageing of our populations but little is known about the burden in low- and middle- income countries. This is a particular concern since low-and middle- income countries, including Latin American and the Caribbean (LAC), are aging more rapidly. At present, over 60% of the world’s population 60 years and older live in these countries and this projection is expected to rise to a high of 80% by 2050 [2].

Age is currently the strongest known predictor of cognitive decline [3, 4]. As such, because of the rapid increase in demographic ageing in all world regions, global prevalence of dementia is predicted to double every 20 years to over 80 million people by 2040 [5]. Since CI has been shown to negatively affect quality of life including functional status [6] countries that are ageing most rapidly will face considerable challenges.

Early life education has been associated with reduced risk of dementia [7, 8] but education influences health in varied ways [9, 10], including by encouraging health-seeking behaviours and healthy lifestyles [9]. The role of education in screening and detection tests has however been controversial since it may influence the ease with which a respondent is able to answer questions asked and display the necessary skills to be measured [11].

As the older population grows in both size and proportion it is important to find out whether the additional years lived are spent with more disease and disability [2]. Health expectancies, which combine information on mortality and morbidity are useful population indicators to assess whether the extra years of life expectancy are in good or poor health [12]. The most widely reported health expectancy is disability-free life expectancy and, while there have been some published studies on mental health expectancies in a number of developed countries [13–18], data on the older population, particularly on cognitive status, is not routinely collected in Latin America and the Caribbean. This paper is the first to report life expectancy with and without CI in LAC, utilising data from a survey conducted in seven LAC countries (Argentina, Barbados, Brazil, Chile, Cuba, Mexico and Uruguay) between the period 1999–2000. In addition we undertake cross-national comparisons to investigate burden of CI at various ages and examine the role education plays in determining cognitive status.

## Method

### Study Design and Sample

The study draws on secondary data derived from The Survey on Health, Well-being and Ageing in Latin America and the Caribbean (SABE). SABE data are freely available from the Inter-university Consortium for Political and Social Research and data was accessed using the search terms ‘ICPSR & SABE & Latin America and the Caribbean.’ SABE, conducted during the period 1999–2000, represents the most comprehensive study conducted in the Region on the elderly population. As a multi-centre project it involved the collection of baseline data in the capital cities of seven countries: Buenos Aires (Argentina), Bridgetown (Barbados), Sao Paolo (Brazil), Santiago (Chile), Havana (Cuba), Mexico City (Mexico) and Montevideo (Uruguay). The project aimed to examine health conditions and functional limitations of persons aged 60 and older in the countries under study and placed special emphasis on those over 80 years old (n = 10,597). The total sample size of the SABE was 10,597 participants. Response rates varied from 85% in Bridgetown, São Paulo, and Mexico City to 60% in Buenos Aires, 84% in Santiago, 95% in Havana, and 66% in Montevideo. Sampling was based on censuses conducted in 1998 in Buenos Aires, 1996 in São Paulo, 1992 in Santiago, 1999 in Havana, 1999 in Mexico City, and 1997 in Montevideo [19]. Surveys were administered to representative samples in each country with samples being drawn from recent census data or from nationally representative surveys [20].

A multistage, clustered sampling with stratification of the units at the highest levels of aggregation was used [20]. The primary sampling unit was a cluster of independent households within predetermined geographic areas grouped into socioeconomic strata and then divided into secondary sampling units, each containing a smaller number of households [20].

Households and target individuals (persons age 60 and older) were randomly selected and target individuals contacted to schedule an interview at home. The interviews were conducted in English, Portuguese or Spanish depending on the official language of the country in which the survey was administered. If a person who agreed to be interviewed failed the cognitive test, a proxy was selected to respond to some parts of the questionnaire [20].

### Definition of CI

The Mini Mental State Examination (MMSE) [21] was used to establish cognitive status in all study samples. The MMSE is a sum-score evaluating various dimensions of cognition (memory, attention and language) and is an index of global cognitive performance [21]. In the SABE Study, a regression analysis had been carried out prior to identify the MMSE questions that would be best to determine cognitive impairment. The Modified MMSE was developed with nine variables instead of the 19 original MMSE variables in a bid to lessen the low literacy bias [22]. Each respondent’s score was then computed, with a score of <12 (out of a maximum of 30) indicating cognitive impairment [20].

The Pfeffer Scale measures functional capacity, taking into consideration ability to perform daily tasks as well as instrumental activities such as conduct of simple fiscal transactions, doing laundry and comprehension of current events and it is used to assess and compare normal and demented adults [23]. The modified MMSE was used in conjunction with the Pfeffer Scale for those persons who obtained a score of 12 or less in the modified MMSE and was administered to an informant or carer accompanying the participant with cognitive deterioration in order to confirm that their level of cognitive decline affected functional capacity [20]. A participant scoring 12 or less on the Modified MMSE and 6 or more on the Pfeffer Scale was considered unable to be interviewed and a proxy of the participant was asked to respond instead [20].

### Level of education

In this study, a high education level was defined as any learning that occurred after completion of secondary school while a low education level was defined as having either primary and/or secondary school education only.

## Statistical Analysis

Logistic regression analysis using Stata version 12 was carried out to assess the relationship of gender, age and education on cognitive status. The Sullivan Method was used to calculate life expectancies with and without CI (CILE and CIFLE) for each country (and gender) by applying country and age-specific CI prevalence from the study outlined above to country-specific life tables. CIFLE reflects the number of remaining years, at a particular age, which an individual can expect to live on average in the absence of CI [24]. Life tables were calculated from mortality rates and population figures obtained from the World Health Organisation. Five year age intervals were used during analysis except for the final age-grouping which was recorded as 85+ years [24]. The curvature of the true survival curve over each age interval (a_x_) was estimated as 0.5 since this is generally a reasonable assumption [24, 25]. The standard error of the estimates of CIFLE was calculated for all age and sex groupings in each country [24]. Men and women were analysed separately. We fitted meta-regression models [26] to all 7 countries together to investigate the relationship between education level and CIFLE at age 60 for men and women separately. The analyses conducted and results presented are unadjusted.

## Results

The mean age of all persons in the study was 70.8 years (SD = 8.2). Overall 58.7% of study participants were female and there were proportionately more females than males in all countries. Around 80% of men and women overall had a low education level though this ranged from around 93% in Barbados (men: 94.4%; women: 92.5%) to 15% in Cuba (men: 14.9%; women: 16.8%). Table 1 shows descriptive statistics for the populations under study in the seven countries.

**Table 1:** Mean Age and Proportion of Males and Females in Study Samples by Country (n = 10,597); Sample size with percentages shown in brackets.

***Country***	***Mean Age (Years)***	***Males (%)***	***Females (%)***	***Low Education Level Males (%)***	***Low Education Level Females (%)***
Argentina (n = 1043)	70.74	383 (37.0)	660 (63.0)	582 (92.5)	322 (85.6)
Barbados (n = 1508)	72.59	924 (60.7)	584 (39.3)	863 (94.4)	527 (92.5)
Brazil (n = 2143)	73.28	881 (40.8)	1262 (59.2)	583 (86.1)	842 (91.5)
Chile (n = 1301)	71.57	446 (34.3)	855 (65.7)	360 (87.6)	678 (92.0)
Cuba (n = 1905)	71.97	708 (38.4)	1197 (61.6)	50 (14.9)	125 (16.8)
Mexico (n = 1247)	64.71	507 (41.2)	740 (58.8)	340 (81.5)	470 (82.7)
Uruguay (n = 1450)	70.94	530 (36.6)	920 (63.4)	395 (77.3)	728 (84.2)
TOTAL	70.8	4379 (41.3)	6218 (58.7)	3173 (80.0)	3692 (78.5)

*Low education level defined as primary and/or secondary school education

**Fig 1:**
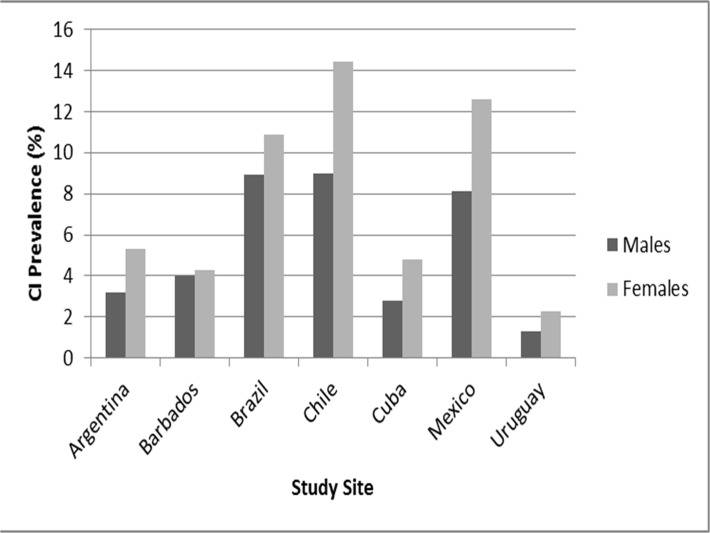
Cognitive Impairment Prevalence by Country and Gender. The cognitive impairment prevalence percentages for each of the seven Latin American and Caribbean countries is shown in this bar graph. Results are stratified by gender.

The prevalence of CI ranged from a low of 8.9% (Argentina) to 21.3% (Barbados) (Fig. 1). A greater proportion of females were recorded as having CI in all countries. Fig. 1 below highlights the prevalence of CI by gender across the study sites.

Logit models fitted to all study sites with the inclusion of a country effect indicated that age, education, gender and country all had a significant impact on cognitive status. For every 1 year increase in age the odds of CI are increased [Odds Ratio (OR) 1.111; 95% CInt (CInt) (1.098–1.126)]. The odds of CI were lower in males when compared to females [Odds Ratio (OR) 0.737; 95% CInt (0.596–0.911)]. Compared to those with a high education level, the odds of CI are increased for those with a low education level [OR 2.926; CInt (1.974–4.337)]. There was a significant difference in the odds of CI between the countries. Compared to Argentina (reference) the odds of CI were significantly higher in Brazil [OR 1.500; CInt (1.007–2.235), Chile [OR 2.398; CInt (1.617–3.558), Mexico [OR 2.948; CInt (1.938–4.485), and Cuba [OR 2.612; CInt (1.569–4.347) and significantly lower in Uruguay [OR 0.372; CInt (0.213–0.650)]. The odds of CI were not significantly different between Barbados and Argentina (reference) [OR 0.871; CInt (0.564–1.345).

Logistic regression analysis was subsequently performed to assess the impact of age, gender and education levels on cognitive status in each country separately. In Argentina, Barbados, Brazil, Chile, Cuba and Mexico the log odds of CI increase linearly as age increases. Thus a one year increase in age increases the odds of CI by 1.120 (95% CInt 1.077–1.165) in Argentina, 1.169 (95%CInt 1.129–1.211) in Barbados, 1.108 (95%CInt 1.081–1.136) in Chile and 1.099 (95%CInt 1.064–1.134) in Cuba. A quadratic age term significantly improved the models for Brazil (p = 0.044) and Mexico (p = 0.008). In Brazil for example, holding gender and education level constant, increasing age from 60 to 61 increases the odds of CI by 1.019 (95% CInt 0.935–1.111) while increasing age from 70 to 71 increases the odds of CI by 1.075 (95% CInt 1.034–1.119) and increasing age from 80 to 81 increases the odds of CI by 1.135 (95% CInt 1.097–1.174). In Mexico, holding gender and education level constant, increasing age from 60 to 61, 70 to 71 and 80 to 81 increases the odds of CI by 0.960 (95% CInt 0.879–1.049), 1.047 (95% CInt 1.011–1.085) and 1.142 (95% CInt 1.083–1.205) respectively. Conversely, the odds of CI decreased with age in Uruguay. The statistically significant quadratic age term (p = 0.039) included in the final regression model for Uruguay showed that an increase in age from 60 to 61, 70 to 71 and 80 to 81 decreases the odds of CI by 1.723 (95% CInt 1.146–2.591), 1.361 (95% CInt 1.122–1.651) and 1.075 (95% CInt 0.976–1.184) respectively.

A significant gender effect was observed in Mexico (p = 0.006) with males having lower odds of CI compared to females after adjusting for age and education level [OR 0.464; 95% CInt (0.268–0.806)].

In Brazil [OR 4.848; 95% CInt (1.173–20.044)], Chile [3.107 (1.098–8.793)], Cuba [2.295 (1.247–4.225)] and Mexico [3.838 (1.368–10.765)], the odds of CI are increased for persons with a low education level when compared to persons with a high education level.

**Fig 2:**
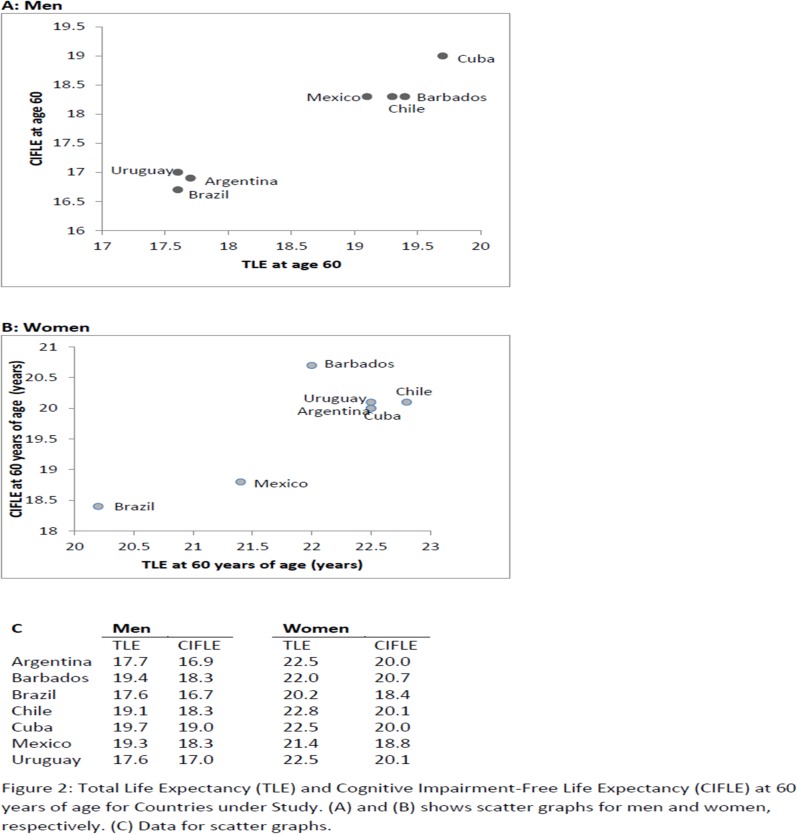
TLE and CIFLE at age 60 by Country and Gender. Total Life Expectancy (TLE) and Cognitive Impairment-Free Life Expectancy (CIFLE) comparisons can be made among each of the seven countries included in the study.

Table 2 presents TLE, CILE, CIFLE and %CIFLE/TLE for males and females by country and at ages 60 and 80 years. At age 60 years, TLE for males varies from a low of 17.6 years (Brazil and Uruguay) to a high of 19.7 years (Cuba). For females, TLE is generally longer than males and at age 60 varied from a high of 22.8 years (Chile) to a low of 20.2 years (Brazil). By the age of 80, the longest TLE and CIFLE for males were observed in Mexico (TLE: 7.2 years, CIFLE: 6.9 years) while the shortest TLE and CIFLE were observed in Barbados (TLE: 6.0 years, CIFLE: 4.8 years). For females at age 80, Chileans and Cubans recorded the longest TLE (8.2 years) while Mexicans recorded the longest CIFLE (7.1 years). See Fig. 2 below.

**Table 2:** TLE, CILE and CIFLE according to age based on MMSE results from the SABE Study (Upper and Lower 95% Confidence Intervals in Brackets).

**Males Aged 60**							
	**Argentina**	**Barbados**	**Brazil**	**Chile**	**Cuba**	**Mexico**	**Uruguay**
**TLE**	17.7	19.4	17.6	19.1	19.7	19.3	17.6
**CILE**	0.8	1.1	0.9	0.8	0.7	1.0	0.8
**CIFLE**	16.9(16.5 17.3)	18.3(18.1 18.6)	16.7(16.4 17.0)	18.3(17.9 18.6)	19.0(18.7 19.3)	18.3(17.9 18.7)	17.0(16.8 17.3)
**%CIFLE/TLE**	95.2	94.5	95.0	95.8	96.6	94.8	97.0
**Females Aged 60**							
**TLE**	22.5	22.0	20.2	22.8	22.5	21.4	22.5
**CILE**	2.5	1.3	1.8	3.1	2.1	2.1	2.0
**CIFLE**	20.0(19.4 20.6)	20.7(20.2 21.1)	18.4(18.1 18.7)	20.1(19.7 20.6)	20.0(19.6 20.4)	18.8(18.2 19.3)	20.1(19.6 20.5)
**%CIFLE/TLE**	88.8	94.1	91.2	88.5	89.0	87.8	89.1
**Males Aged 80**							
**TLE**	6.3	6.0	6.8	6.8	6.8	7.2	6.5
**CILE**	0.1	1.2	0.8	0.2	0.5	0.3	0.5
**CIFLE**	6.2(5.8 6.5)	4.8(4.5 5.2)	6.2(6.0 6.5)	6.6(6.3 6.9)	6.3(6.0 6.6)	6.9(6.4 7.3)	6.0(5.6 6.4)
**%CIFLE**	98.4	80.2	91.3	97.0	93.2	95.0	92.9
**Females Aged 80**							
**TLE**	7.8	7.5	7.2	8.2	8.2	7.8	7.9
**CILE**	0.7	1.2	1.2	1.5	1.5	0.7	1.0
**CIFLE**	6.4(5.8 7.1)	6.7(6.3 7.2)	6.0(5.6 6.3)	6.7(6.2 7.2)	6.7(6.2 7.1)	7.1(6.6 7.7)	6.9(6.4 7.3)
**%CIFLE**	82.4	89.4	82.8	82.1	81.2	91.3	87.0

At age 60, males in Uruguay (97.0%) and Cuba (96.6%) spend the greatest proportion of their remaining lives free of CI when compared to those at other study sites despite males in Uruguay having the shortest TLE at age 60. Among females aged 60, the greatest proportion of life is spent free of CI in Barbados (94.1%) and Brazil (91.2%). At age 80, males in Argentina (98.4%) and Chile (97.0%) and females in Mexico (91.3%) and Barbados (89.4%) spend the greatest proportion of their lives free of CI.

To see the extent to which educational level in a country explained the observed differences in CIFLE we fitted meta-regression models to CIFLE at age 60 separately for males and females but found no association (males: 0.408; females: 0.695). This conclusion remained when the analysis was repeated excluding Cuba which had by far the largest percentage with high education (S1 and S2 Figs).

## Discussion

In the LAC countries under study, the odds of CI increased with age in all countries except Uruguay. A significant gender effect was observed only in Mexico with males having lower odds of CI compared to females. For males TLE at age 60 years was highest in Cuba and lowest in Brazil and Uruguay. CIFLE at age 60 for males was highest in Barbados, Chile and Mexico and lowest in Brazil. However, although Uruguay had the lowest TLE and did not have the highest CIFLE at age 60, Uruguayan men experienced the greatest proportion of their remaining life free of CI (97.0%). For females at age 60 years, TLE was highest in Chile and lowest in Brazil. CIFLE for females at age 60 was highest in Barbados and lowest in Brazil. Although higher odds of CI were observed in those with low education in Brazil, Chile, Cuba and Mexico, level of education did not appear to explain the differences in CIFLE at age 60 in LAC countries.

The differences observed among countries in our study are noteworthy and future research is necessary to determine the exact cause. Possible explanations include the effect of confounding factors not controlled for such as race/ethnicity, or differences in the application of the survey instrument at each study site. In addition, published research indicates that among the study countries there is great variation in the structure of the healthcare systems and the role played by the public health sector in protecting the health of the older population and these could account for the differences observed between countries [27].

Worth noting is that while the Uruguay sample had a higher mean age than Mexico (70.94 and 64.71 respectively), TLE at age 60 in Mexico is much higher. This may be explained by the oversampling of the target population in Mexico where all eligible individuals found in a target home were interviewed compared to Uruguay where only one individual was selected per household [20]. It is also possible that in Uruguay stratification and subsequent sampling was defined by socioeconomic as well as geographic and global indicators while in Mexico stratification may have been based solely on geography [27]. Reports on cognitive impairment of older people in developing countries are rare. However, the Mexican Health Ageing Study has produced data estimating prevalence of CI (no dementia) that can be used for comparison with results obtained in the present study. Published data from the Mexican Study estimates CI prevalence at 28.7% [28]. These estimates are higher than those obtained in the present study for the Mexican population (approximately 10.8%) and may reflect the fact that SABE data was collected from city dwellers only who may have greater access to health care, belong to higher socio-economic groupings and have a healthier diet. Additionally, differing methodologies used for diagnosis of CI may have led to variations in results.

Studies on dementia-free life expectancy (DFLE) for some Latin American countries (Uruguay, Chile and Brazil) have been published and estimates range from 3.4% to 7.1% [28]. The results of the present study provide CI prevalence estimates in these countries between 1.9% and 12.5%.

Comparisons with developed countries such as Australia, Canada and England and Wales provide much needed perspective. An analysis of DFLE in Australia during the period 2004–2006 revealed that males and females aged 65 were expected to live a further 18.3 and 21.5 years respectively [14]. Of these years, males could expect to have a DFLE of 17.1 years and females 19.5 years [14]. Figures for the Canadian population are similar, Canadian males and females aged 65 are expected to live a further 16.4 years and 19.4 years respectively. Of these years, 13.8 years for males and 17.2 years for females are expected to be lived free from CI [18]. When compared to Australian and Canadian males, TLE and CIFLE are lower in all LAC countries. For example in Cuba, the country with the highest TLE and CIFLE at age 65, a male is expected to live a further 15.9 years of which 15.3 would be spent CI-free. When comparisons are made among females, the TLE and CIFLE for Australian females are higher than those in Canada but LAC countries record the lowest estimates. In Australia, a female aged 65 is expected to live a further 21.5 years of which 19.5 would be spent free of CI while in Chile, the LAC country with the highest TLE at age 65, a female is expected to live a further 18.7 years of which 16.2 would be spent CI-free.

The Medical Research Council Cognitive Function and Ageing Study (MRC CFAS) allows for comparisons between LAC countries and the older population in England and Wales in 1991 [29]. In both settings, men live shorter lives than women. Among both sexes, TLE is higher across all age-groups in LAC countries when compared to that observed in England and Wales. For instance, males in Cuba and Barbados at age 65 can expect to live a further 15.9 and 15.7 years respectively while those in England and Wales can expect to live a further 14.1 years. In the case of females at 65 years of age those in England and Wales have a TLE of 17.8 years and a CILE of 1.2 years. This is lower than the CILE for the same age in all LAC study countries. It must be noted though these comparisons are based on estimates over a decade apart (CFAS:1991, LAC 1999–2000) [29].

The effect of education on health outcomes including cognitive impairment has been published in relation to previous studies [10, 30]. The results of this study, which show that the odds of CI are greater in persons with low education levels, support previous research from the United Kingdom which concluded that there is a substantial burden of life expectancy with CI for groups with low education [10, 30].

Among the limitations of this current study is the inability to disaggregate CI by type of dementia. However, SABE represents the only comprehensive cross national study on the health of elders in the LAC region and while likely to have underestimated prevalence estimates due to its focus on city dwellers, its results are useful. Secondly, no data were collected from adults living in institutions in any of the study countries and so may not be representative of that population. Research indicates though that the percentage of the population in institutions in LAC countries is quite small and so this bias is also likely to be small [31].

The main strength of this study is the fact that CI and CI free life expectancies have not been published for such a wide range of LAC countries prior to this and provide useful data for policy makers to assess healthy ageing trends.

## Conclusion

The results from this study are consistent with other published findings which have indicated that age, sex and education level are significant predictors of cognitive status. Novel though is the comparison of healthy life expectancies among Latin American and Caribbean countries and these show substantial differences in the absolute years lived and the proportion of remaining life spent free of cognitive impairment. These results provide a greater understanding of the burden of CI in Latin America and the Caribbean and highlight the need for follow-up surveys to be conducted as their populations continue to age.

## Supporting Information

S1 Fig Meta-regression Results—Relationship between Education Level and CIFLE at Age 60 Males. Shows the extent to which educational level in a country explained the observed differences in Cognitive Impairment-Free Life Expectancy (CIFLE) at age 60 separately for males.
(TIF)

S2 Fig Meta-regression Results—Relationship between Education Level and CIFLE at Age 60 Females. Shows the extent to which educational level in a country explained the observed differences in Cognitive Impairment-Free Life Expectancy (CIFLE) at age 60 separately for females.
(TIF)
