# Supplementary figures and images for: Life Expectancy with and without Cognitive Impairment in Seven Latin American and Caribbean Countries
Source: PLoS One. 2015 Mar 23;10(3):e0121867. doi: 10.1371/journal.pone.0121867 (PMC4370415; doi:10.1371/journal.pone.0121867)

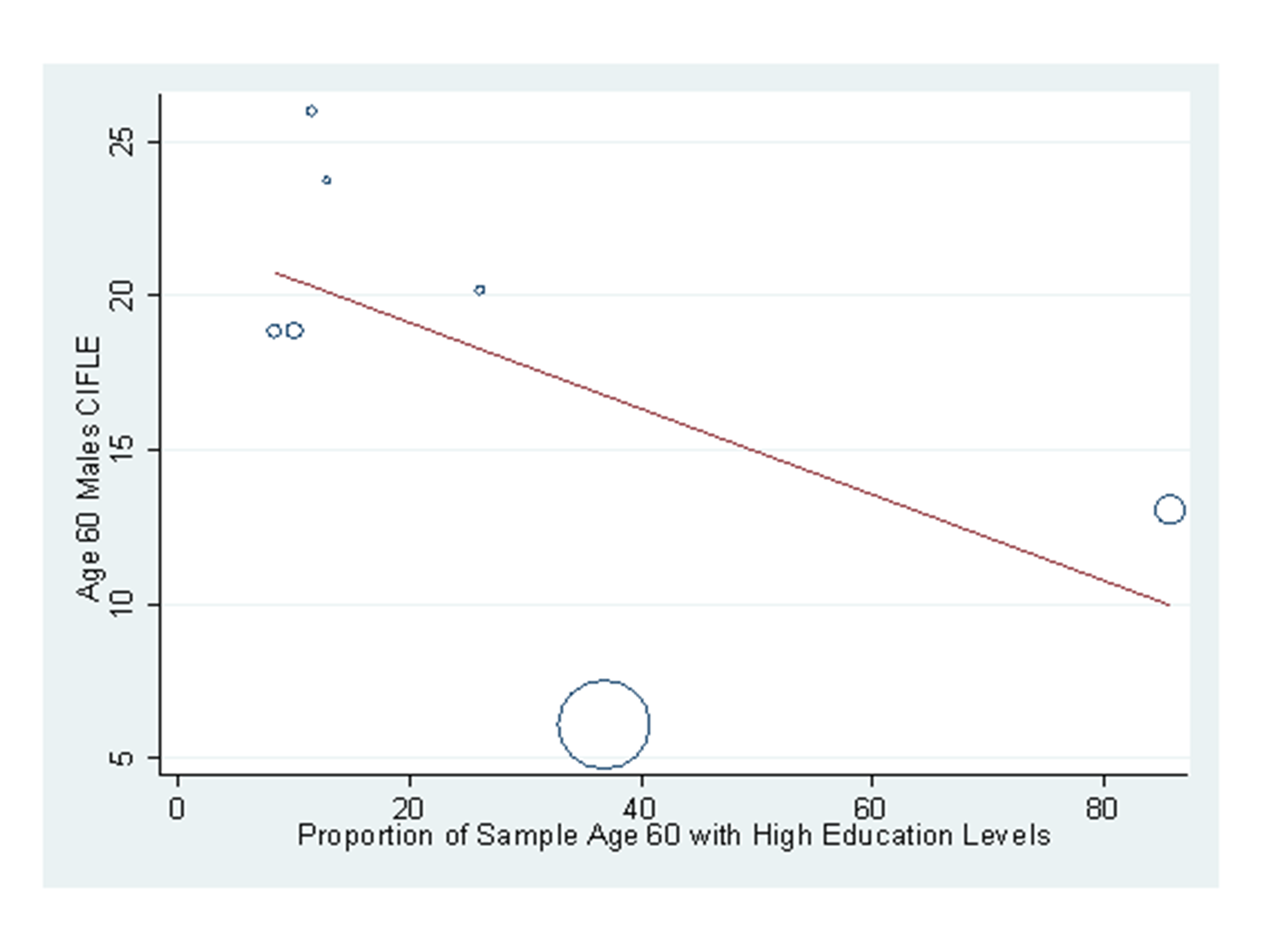

Supplement: S1 Fig — Shows the extent to which educational level in a country explained the observed differences in Cognitive Impairment-Free Life Expectancy (CIFLE) at age 60 separately for males. (TIF) [file pone.0121867.s001.tif]

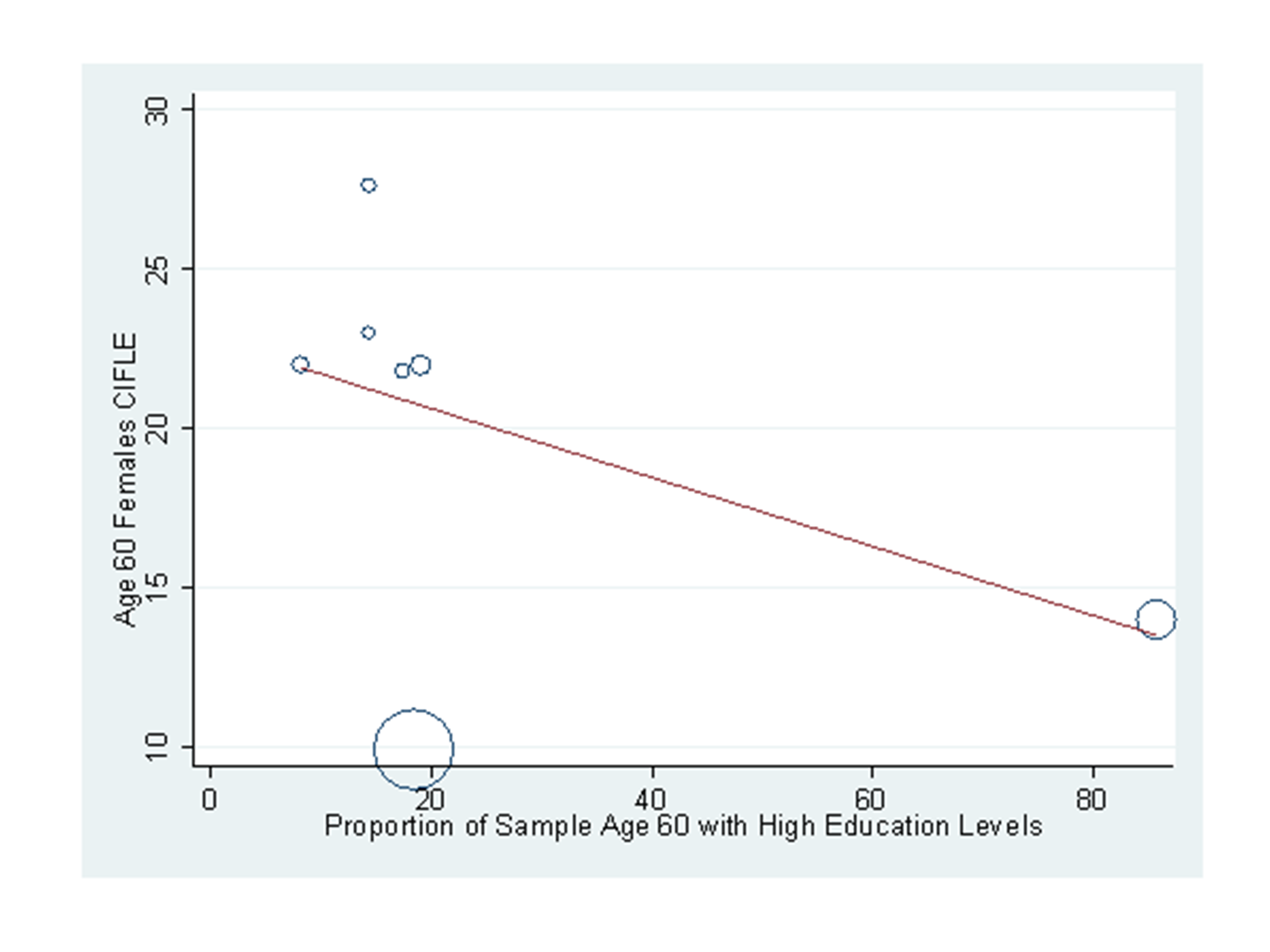

Supplement: S2 Fig — Shows the extent to which educational level in a country explained the observed differences in Cognitive Impairment-Free Life Expectancy (CIFLE) at age 60 separately for females. (TIF) [file pone.0121867.s002.tif]
